# Supplementary material for: Vigna radiata extracts in pumpkin and soya bean oil: A novel therapeutic approach for Alzheimer’s disease
Source: PLoS One. 2025 Apr 15;20(4):e0321183. doi: 10.1371/journal.pone.0321183 (PMC11999159; doi:10.1371/journal.pone.0321183)
Supplement: S1 File — (DOCX) [file pone.0321183.s001.docx]

Supporting information

**Result of VRSO and VRPO on cognitive mapping and memory impairment in MWM task Mean values** **to generate graph**

|  | Normative | | | AD like phenotype | | | Standard Care group | | | VRPO 250 mg/kg | | | VRPO 500 mg/kg | | | VRSO 250 mg/kg | | | VRSO 500 mg/kg | | |
| --- | --- | --- | --- | --- | --- | --- | --- | --- | --- | --- | --- | --- | --- | --- | --- | --- | --- | --- | --- | --- | --- |
| North Quadrant | 20.0 | 19. | 20. | 40. | 45. | 41. | 26. | 23. | 25. | 30. | 33. | 32. | 22. | 26. | 23. | 30. | 31. | 32. | 30. | 20. | 22. |
| South Quadrant | 22.0 | 21. | 20. | 42. | 43. | 40. | 25. | 26. | 24. | 28. | 33. | 31. | 23. | 24. | 22. | 34. | 35. | 33. | 34. | 22. | 20. |
| East Quadrant | 23.0 | 22. | 20. | 43. | 45. | 43. | 27. | 25. | 26. | 29. | 30. | 32. | 24. | 24. | 25. | 34. | 33. | 31. | 34. | 21. | 25. |
| West Quadrant | 19.5 | 20. | 21. | 44. | 45. | 40. | 25. | 26. | 27. | 30. | 33. | 29. | 22. | 21. | 25. | 30. | 32. | 30. | 30. | 22. | 21. |

**Mean values to generate graph Effect of VRPO and VRSO on exploratory and anxiety like behavior in elevated plus maze task**

|  | Normative | | | AD like phenotype | | | Standard Care group | | | VRPO 250 mg/kg | | | VRPO 500 mg/kg | | | VRSO 250 mg/kg | | | VRSO 500 mg/kg | | |
| --- | --- | --- | --- | --- | --- | --- | --- | --- | --- | --- | --- | --- | --- | --- | --- | --- | --- | --- | --- | --- | --- |
| Transfer Latency day 27 | 0.30 | 0.40 | 0.25 | 1.4 | 1.3 | 1.5 | 0.4 | 0.50 | 0.45 | 0.4 | 0.50 | 0.45 | 0.3 | 0.29 | 0.32 | 0.4 | 0.44 | 0.45 | 0.24 | 0.22 | 0.19 |
| Transfer Latency day 28 | 0.25 | 0.16 | 0.18 | 1.1 | 1.2 | 0.9 | 0.4 | 0.38 | 0.35 | 0.4 | 0.38 | 0.35 | 0.2 | 0.28 | 0.27 | 0.4 | 0.40 | 0.37 | 0.20 | 0.18 | 0.17 |

**Effect of VRPO and VRSO on fear conditioning and exploration in passive avoidance task**

**Mean values to generate graph**

|  | Normative | | | AD like phenotype | | | Standard Care group | | | VRPO 250 mg/kg | | | VRPO 500 mg/kg | | | VRSO 250 mg/kg | | | VRSO 500 mg/kg | | |
| --- | --- | --- | --- | --- | --- | --- | --- | --- | --- | --- | --- | --- | --- | --- | --- | --- | --- | --- | --- | --- | --- |
| Step down latency | 4. | 5. | 4.4 | 1. | 0.5 | 1.1 | 3. | 4. | 4. | 2.5 | 3. | 4. | 4. | 5. | 3.5 | 2.5 | 4. | 5. | 5. | 4. | 4.5 |

**Effect of VRPO and VRSO on fear conditioning and exploration on head dipping task.** **Mean values to generate graph**

| Normative | | | AD like phenotype | | | Standard Care group | | | VRPO 250 mg/kg | | | VRPO 500 mg/kg | | | VRSO 250 mg/kg | | | VRSO 500 mg/kg | | |
| --- | --- | --- | --- | --- | --- | --- | --- | --- | --- | --- | --- | --- | --- | --- | --- | --- | --- | --- | --- | --- |
| 13. | 12. | 14. | 3. | 3. | 4. | 7. | 9. | 10. | 8. | 9. | 10. | 11. | 12. | 13. | 10. | 12. | 10. | 14. | 13. | 13. |

**Effect of VRPO and VRSO on wire hanging task.** **Mean values to generate graph**

| Normative | | | AD like phenotype | | | Standard Care group | | | VRPO 250 mg/kg | | | VRPO 500 mg/kg | | | VRSO 250 mg/kg | | | VRSO 500 mg/kg | | |
| --- | --- | --- | --- | --- | --- | --- | --- | --- | --- | --- | --- | --- | --- | --- | --- | --- | --- | --- | --- | --- |
| 1. | 1.2 | 1.5 | 0.3 | 0.4 | 0.25 | 1. | 0.70 | 1. | 0.9 | 1. | 1.2 | 1. | 1.3 | 1.5 | 0.98 | 1. | 1.1 | 1.2 | 1.5 | 1.4 |

**Acetyl-cholinesterase inhibitory effect of VRPO and VRSO treatment, Mean values to generate graph**

| Normative | | | AD like phenotype | | | Standard Care group | | | VRPO 250 mg/kg | | | VRPO 500 mg/kg | | | VRSO 250 mg/kg | | | VRSO 500 mg/kg | | |
| --- | --- | --- | --- | --- | --- | --- | --- | --- | --- | --- | --- | --- | --- | --- | --- | --- | --- | --- | --- | --- |
| 3.5 | 3.0 | 4.0 | 9.0 | 10.00 | 8. | 4.00 | 5. | 5.5 | 5. | 4.0 | 6. | 4.0 | 3.0 | 3.50 | 4. | 5.0 | 4.0 | 3.0 | 3.5 | 3. |

**Effect of VRPO and VRSO on mRNA expression of SFRP4, neurodegenerative and neuro-inflammatory biomarkers Mean values to generate graph**

|  | Normative | | | AD like phenotype | | | Standard Care group | | | VRPO 250 mg/kg | | | VRPO 500 mg/kg | | | VRSO 250 mg/kg | | | VRSO 500 mg/kg | | |
| --- | --- | --- | --- | --- | --- | --- | --- | --- | --- | --- | --- | --- | --- | --- | --- | --- | --- | --- | --- | --- | --- |
| SFRP4 | 1. | 1.30 | 1.40 | 4. | 5.0 | 6.0 | 3.0 | 2.4 | 2.3 | 2.6 | 2.0 | 2.4 | 1.4 | 1.5 | 1.6 | 2. | 2.5 | 2.6 | 1.20 | 1.6 | 1.15 |
| IL-1α | 1. | 0.80 | 0.90 | 3. | 4.0 | 5.0 | 4.0 | 3.0 | 2.0 | 3.0 | 2.0 | 1.4 | 1.4 | 2.0 | 1.0 | 3. | 2.0 | 1.4 | 1.00 | 1.4 | 1.00 |
| IL-1β | 1. | 1.10 | 1.20 | 3. | 4.4 | 4.0 | 4.4 | 2.0 | 2.3 | 2.0 | 2.3 | 1.4 | 1.4 | 2.2 | 1.3 | 2. | 2.3 | 1.4 | 0.90 | 1.4 | 1.10 |
| β-Secretase | 1. | 0.98 | 0.99 | 4. | 5.0 | 6.0 | 5.0 | 3.0 | 2.0 | 3.0 | 2.0 | 1.9 | 1.9 | 2.1 | 1.4 | 3. | 2.0 | 1.9 | 1.10 | 1.9 | 1.20 |
| ABPP | 1. | 1.20 | 1.10 | 5. | 4.0 | 3.6 | 4.0 | 2.0 | 2.0 | 2.0 | 2.2 | 1.5 | 1.5 | 1.9 | 1.6 | 2. | 2.2 | 1.5 | 1.20 | 1.5 | 1.40 |
| AchE | 1. | 1.30 | 1.10 | 4. | 3.0 | 5.0 | 3.0 | 2.0 | 1.9 | 2.0 | 2.0 | 2.4 | 2.4 | 1.7 | 1.8 | 2. | 2.0 | 2.4 | 1.30 | 2.4 | 1.10 |
| TNF-α | 1. | 1.20 | 1.30 | 4. | 4.0 | 4.0 | 4.0 | 2.0 | 1.9 | 2.0 | 3.0 | 1.4 | 1.4 | 1.6 | 1.9 | 2. | 3.0 | 1.4 | 1.00 | 1.4 | 0.96 |
| IL-6 | 1. | 0.90 | 0.98 | 4. | 5.0 | 6.0 | 3.5 | 3.0 | 1.0 | 3.0 | 2.0 | 1.3 | 1.3 | 1.4 | 1.7 | 3. | 2.0 | 1.3 | 0.97 | 1.3 | 1.20 |
