# Supplementary material for: Vigna radiata extracts in pumpkin and soya bean oil: A novel therapeutic approach for Alzheimer’s disease
Source: PLoS One. 2025 Apr 15;20(4):e0321183. doi: 10.1371/journal.pone.0321183 (PMC11999159; doi:10.1371/journal.pone.0321183)
Supplement: Attachment — Submitted filename: comments new haroon.docx [file pone.0321183.s003.docx]

**Response Letter to Reviewer’s Comments**

**Manuscript Draft ID:** PONE-D-24-45075R1

**Title:** *Vigna radiata* Extracts in Pumpkin and Soya Bean Oil: A Novel Therapeutic Approach for Alzheimer's disease

**Journal:** PLOS ONE

Dear Editor,

We are very grateful to the reviewers for their appropriate and constructive suggestions and for their proposed corrections to improve the paper. We have addressed all the issues raised and have modified the paper accordingly. Please find attached an amended version of the manuscript with changes highlighted. Below is a summary of the changes we performed and our responses to the reviewers’ comments and recommendations.

**Reviewer #1:**

**To comply with PLOS ONE submissions requirements, in your Methods section, please provide information on inhalant anesthesia used in Euthanasia procedure**

**Response: To comply with PLOS ONE submission requirement, in method section information about anesthesia and euthanasia is added and highlighted.**
